# Attitudes and Barriers Toward Consumption of More Plant-Based Foods Among Danish Patients with Celiac Disease

**DOI:** 10.3390/nu18111673

**Published:** 2026-05-23

**Authors:** Christina Chinchay Nielsen, Allan Linneberg, Line Lund Kårhus, Signe Ulfbeck Schovsbo, Nikita Misella Hansen

**Affiliations:** 1 Center for Clinical Research and Prevention, Copenhagen University Hospital—Bispebjerg and Frederiksberg, 2000 Copenhagen, Denmark; christina.chinchay.nielsen@regionh.dk (C.C.N.); signe.ulfbeck.schovsbo@regionh.dk (S.U.S.); nikita.misella.hansen@regionh.dk (N.M.H.); 2 Department of Clinical Medicine, Faculty of Health and Medical Sciences, University of Copenhagen, 2200 Copenhagen, Denmark

**Keywords:** celiac disease, gluten-free diet, plant-dominant diet, plant-based foods, attitudes, barriers, adherence

## Abstract

**Background**: Celiac disease (CeD) requires lifelong adherence to a gluten-free diet (GFD). However, there is evidence that a GFD may lead to an unhealthy cardiometabolic risk profile and potentially increase the risk of cardiovascular disease in some patients. Incorporating plant-based foods (primarily derived from plants) into a GFD may offer a solution to improve cardiometabolic health. Thus, this study aimed to identify the attitudes toward and barriers to adopting a more plant-dominant diet among Danish patients with CeD. **Methods**: A cross-sectional survey was distributed to 2861 members of the Danish Celiac Society. Data from 959 patients with confirmed CeD were included. **Results**: Most participants (58.5%) reported adapting their diet after diagnosis by combining gluten-free products with plant-based foods, while 31.2% relied solely on gluten-free replacements. Dietary adaptation was primarily shaped by the limited availability of gluten-free plant-based foods (64%), taste/texture (55%), and cost (51%). More than half of the patients (56.8%) considered ‘eating more plant-based foods’, with ‘health’ being the primary motivator (70%), followed by ‘climate’ (50%) and ‘taste’ (36%). However, several barriers to a more plant-dominant diet were identified. Most notably, ‘taste and texture’ (71%), ‘limited availability of gluten-free plant-based foods’ (68%), ‘nutritional concerns’ (56%), and ‘cost’ (54%) were reported as barriers. **Conclusions**: Most Danish patients with CeD were generally positive about increasing their intake of plant-based foods; however, barriers to such dietary changes remain. Ongoing follow-up, practical guidance from dietitians, and accessible evidence-based resources may help patients maintain a nutritionally balanced, plant-dominant GFD that supports long-term health.

## 1. Introduction

Celiac disease (CeD) is an autoimmune disease that develops in genetically susceptible individuals upon ingestion of gluten, a protein found in wheat, barley, and rye. Gluten exposure activates both adaptive and innate immune responses in the small intestine, leading to inflammation and villous atrophy in the intestine. Villous atrophy substantially reduces the absorptive surface area, resulting in an immature and functionally impaired epithelium. This structural damage disrupts the absorption of macronutrients and micronutrients. These changes can lead to malabsorption, weight loss, failure to thrive, and micronutrient deficiency [1].

The clinical manifestations of CeD vary widely. Gastrointestinal symptoms include chronic diarrhea, abdominal distention, abdominal pain, and bloating, which reflect underlying intestinal inflammation [2]. Extraintestinal manifestations, such as iron-deficiency anemia, dermatitis herpetiformis, chronic fatigue, osteoporosis, and increased liver transaminases are common and further contribute to the diagnostic complexity of CeD [1,2].

A strict lifelong gluten-free diet (GFD) is currently the only effective treatment for achieving mucosal healing and symptom remission [1]. However, gluten-containing cereals are important dietary sources of proteins, fibers, and micronutrients. Thus, adherence to a GFD may contribute to nutritional inadequacy [3]. Several studies have shown that individuals on a GFD often consume less fiber and more saturated fat and sugar, partly due to the exclusion of whole grains and increased consumption of industrially produced gluten-free substitutes [4,5,6,7,8]. These dietary patterns may contribute to deficiencies in essential micronutrients such as iron, magnesium, folate, and B vitamins [8]. Additionally, long-term adherence to a GFD has been associated with adverse alterations in lipid and glucose metabolism, including increased total cholesterol and fasting glycemia [9,10,11].

These metabolic changes may further contribute to a less favorable cardiometabolic profile [12]. Dietary composition and metabolic responses are associated with cardiometabolic health; therefore, concerns have emerged that the nutritional limitations of a typical GFD may have implications for cardiovascular risk [1,10,13]. A large prospective analysis of the UK Biobank revealed that individuals with CeD had a higher incidence of cardiovascular disease (CVD) than those without CeD. This increased risk was observed despite the CeD group having a more favorable baseline risk profile, including a lower body mass index (BMI), lower smoking rates, and lower blood pressure. Importantly, this association remained significant after adjusting for traditional cardiovascular risk factors [14,15]. Similar findings from large population-based studies in Sweden and Finland have also shown a higher incidence of CVD among individuals with CeD [16,17]. This has led researchers to propose that the nutritional composition of the GFD, particularly its low fiber content and high intake of saturated fat, salt, and sugar, contributes to long-term cardiometabolic risk [12,18,19,20].

Therefore, improving the nutritional quality of a GFD is an important research area. In the general population, plant-based dietary patterns (typically defined as dietary patterns that prioritizes the consumption of foods derived from plants, while minimizing animal-products) are consistently associated with a reduced risk of CVD and improved metabolic health [21,22,23,24,25]. Incorporating more minimally processed, naturally gluten-free plant foods, such as vegetables, legumes, fruits, nuts, and seeds, can increase fiber and micronutrient intake. This may, in turn, help address common deficiencies in CeD and limit the intake of saturated fats, thereby providing a more well-balanced whole-food approach for patients with CeD.

Despite the potential of plant-dominant diets, evidence is limited on how individuals with CeD perceive the benefits and barriers of plant-based foods or how different factors shape their dietary choices. Therefore, this study aimed to explore Danish patients with CeD attitudes toward and perceived barriers to increasing their intake of plant-based foods.

In this study, plant-based foods refer to the definition provided to survey participants (detailed in Section 2. Materials and Methods). By contrast, we define ‘plant-dominant diet’ as a gluten-free dietary pattern in which naturally gluten-free, minimally processed plant foods (e.g., vegetables, legumes, fruits, nuts, seeds) provide a larger proportion of total intake, without necessarily excluding animal products.

## 2. Materials and Methods

A cross-sectional questionnaire survey was conducted on Danish individuals. From 3 March to 19 March 2025, the survey was distributed to 2861 members of the Danish Celiac Society using an online survey software, Enalyzer (https://www.enalyzer.com). All the responses were anonymized. A total of 984 participants completed the questionnaire.

The use and processing of data were approved and registered by the Local Data Authority in the Capital Region of Denmark (P-2025-19468) on 26 August 2025. In accordance with Danish regulations, questionnaire surveys do not require ethical approvals.

### 2.1. Survey and Data Collection

Data were collected using a structured questionnaire covering diagnostic history, dietary habits, and attitudes and barriers related to the consumption of plant-based foods.

In the survey, plant-based foods were defined as foods primarily derived from plants, including fruits, vegetables, legumes, nuts, seeds, and whole grains. The definition also encompassed plant-based food products and plant-based beverages. Participants were informed that eating plant-based does not necessarily imply following a vegetarian or vegan diet, but may also reflect a more flexible dietary pattern, in which plant-based foods constitute a greater proportion of the diet. This definition reflects a broad food-level framework for the survey and includes both minimally processed plant-foods and plant-based products. It does not correspond to a specific dietary pattern such as vegan and vegetarian diets or a plant-dominant diet.

The survey comprised 32 questions in four sections: (1) participant characteristics (including demographics), (2) dietary habits and experiences of following a GFD, (3) attitudes towards plant-based foods, and (4) motivations and barriers to consuming more plant-based foods while following a GFD. All questions in the survey were not mandatory, and participants could skip items if they preferred not to answer.

Most of the survey was designed using single-select questions that required participants to choose one answer from two to eight, predefined options. The survey also included multiple-response questions and ranking questions, in which participants selected their top three priorities from a list of five to nine options.

A 5-point Likert scale was used to capture participants’ perceptions and experiences along a spectrum, assessing aspects such as changes in time spent planning meals or the perceived difficulty of consuming a more balanced diet.

Contingency questions were included in the survey to ensure that only relevant follow-up questions were displayed based on the prior responses.

### 2.2. Statistical Analysis

The survey data were exported to R (version 4.5.0; R Foundation for Statistical Computing, Vienna, Austria) for analysis. Descriptive statistics were used to summarize the participants’ characteristics and survey responses. For each question, complete-case data were used. Because the overall distribution of responses remained consistent despite variation in item-level denominators, additional sensitivity analyses were not performed.

Spearman’s rho was applied to assess correlations between duration of CeD and dietary habits. Differences in responses across age groups were tested using the chi-square test or Fisher’s exact test when expected counts were <5.

Participants were stratified based on whether they had considered consuming more plant-based foods (yes/no), and differences in response patterns between the groups were analyzed. A multivariable logistic regression model including categorical predictors age, sex, disease duration, and dietetic counselling, was conducted to examine independent predictors associated with having considered eating more plant-based foods. Odds ratios and 95% confidence intervals were calculated. *p*-values for age groups and disease duration were obtained using Wald tests. Statistical significance was set at *p* ≤ 0.05.

## 3. Results

### 3.1. Characteristics of Patients with CeD

Of the 2861 individuals invited to participate, 984 responded to the questionnaires. Of the total responses, 959 had a confirmed diagnosis of CeD and were included in the analysis. The participants’ characteristics are presented in Table 1.

Approximately half of the patients had a CeD diagnosis for more than ten years prior to receiving the questionnaire (47.1%). A total of 664 patients (69.7%) were diagnosed in a hospital. In addition, 756 (79.7%) patients were referred to a dietitian.

### 3.2. Factors Influencing Attitudes and Barriers Towards Eating More Plant-Based Foods Among Patients with CeD

A summary of the patients’ dietary habits is presented in Table 2.

**Figure 1:**
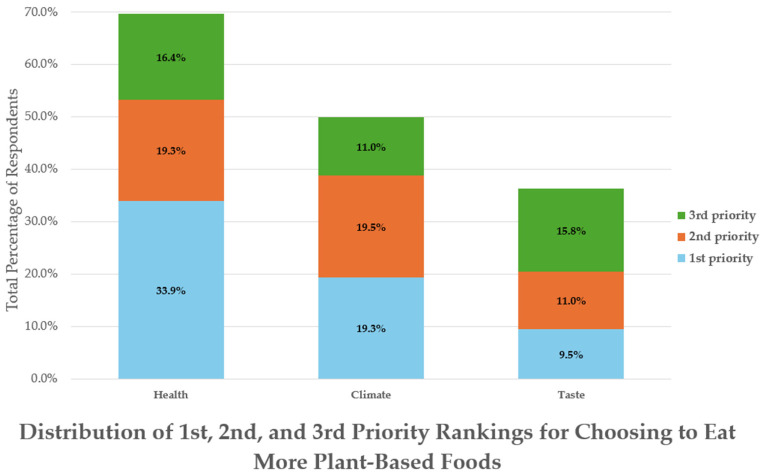
Distribution of prioritized factors influencing celiac disease patients’ choice to follow a more plant-dominant diet while on a gluten-free diet. Patients selected their top three priorities, and bars indicate the ranking of each item as patient’s 1st, 2nd, and 3rd priority. Y-axis represents the total percentage of patients who completed the ranking question (n = 507).

**Figure 2:**
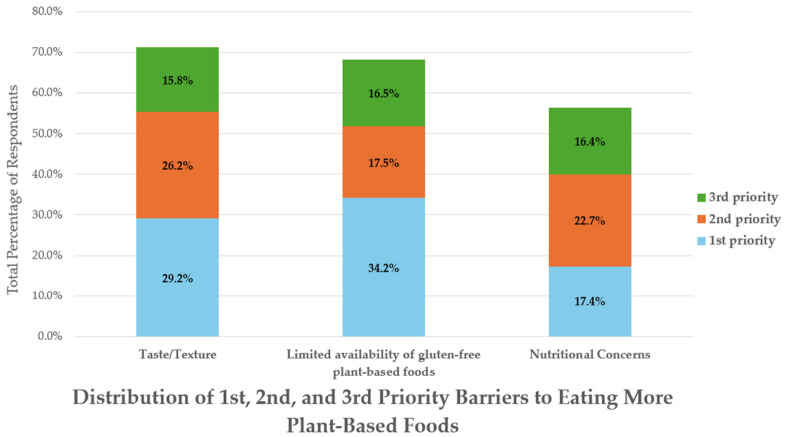
Distribution of prioritized barriers toward eating a more plant-dominant diet while following a gluten-free diet among patients. Patients selected their top three priorities, and bars indicate the ranking of each item as patient’s 1st, 2nd, and 3rd priority. Y-axis represents the total percentage of patients who completed the ranking question (n = 874).

Most patients (56.8%) considered increasing their intake of plant-based foods (Table 2). Figure 1 shows the distribution of 1st, 2nd, and 3rd priority rankings for choosing a more plant-dominant dietary approach while following a GFD. When combining these rankings, ‘health’ was the most frequently selected priority (69.6%; n = 353), followed by ‘climate’ (49.9%; n = 253) and ‘taste’ (36.3%; n = 184) (Figure 1; full ranking distributions for patients who had not considered eating more plant-based foods are shown in Supplementary Figure S1). Figure 2 shows the distribution of 1st, 2nd, and 3rd priority rankings for barriers for choosing a more plant-dominant dietary approach diet while following a GFD. ‘Taste/texture’ was the most frequently selected barrier overall (71.2%), followed by ‘limited availability of gluten-free plant-based foods’ (68.2%) and ‘nutritional concerns’ (56.4%).

After diagnosis, 58.5% of patients reported changing their diet by combining gluten-free products with plant-based food. In comparison, 31.2% of patients reported changing their diet exclusively through gluten-free replacements (Table 2).

**Figure 3:**
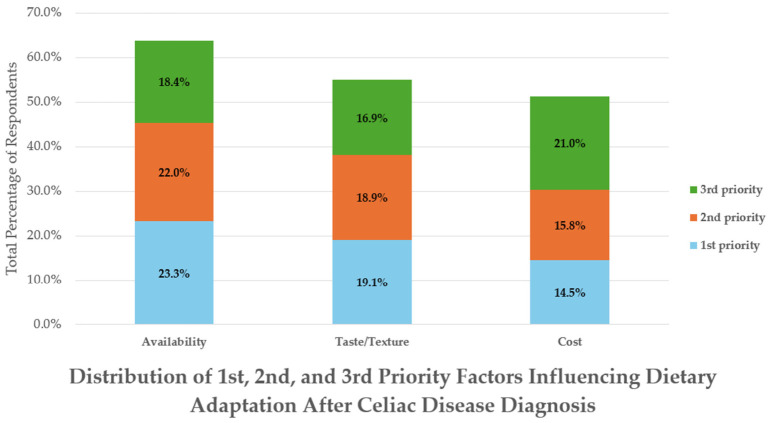
Distribution of responses according to the factors influencing how patients adapted their current diet after celiac disase diagnosis. Patients selected their top three priorities, and bars indicate the ranking of each item as patient’s 1st, 2nd, and 3rd priority. Y-axis represents the total percentage of patients who completed the ranking question (n = 904).

Figure 3 shows the distribution of 1st, 2nd, and 3rd priority rankings for factors influencing dietary adaptation after CeD diagnosis. ‘Limited availability of gluten-free plant-based foods’ followed by ‘taste/texture’, and ‘cost’ were the top three prioritized factors (63.7%, 55.0%, and 51.3%, respectively). Notably, ‘limited availability of gluten-free plant-based foods’ received the highest proportions of first- and second-rank selections among the top three factors (23.3% [n = 211] and 22.0% [n = 199], respectively), while ‘cost’ ranked third overall (21.0%) (Figure 3). For additional prioritized factors, see Supplementary Figures S2–S4.

In the multivariable logistic regression model (Table 3; Supplementary Table S1) patients < 18 years, compared to all other age groups had significantly lower odds of having considered increasing their plant-based intake (*p* < 0.001). Males had lower odds than females (OR = 0.52, 95% CI 0.36–0.74, *p* < 0.001). In contrast neither disease duration (*p* = 0.78) nor prior dietetic counselling (OR = 0.87, 95% CI 0.62–1.22, *p* = 0.41) were significantly associated with the outcome. Details on data across all category groups are provided in Supplementary Table S1.

### 3.3. Dietary Habits and Their Associations with Age and Disease Duration in Patients with CeD

Spearman’s rho indicated weak negative correlations between disease duration and (i) the perceived difficulty finding gluten-free foods that match dietary preferences (r = −0.09; 95% CI −0.16, −0.03) and (ii) the relative time spent planning meals (r = −0.27; 95% CI −0.33, −0.21). No statistically significant correlation was observed between cooking habits and disease duration (r = 0.04; 95% CI −0.02, 0.11).

Across age groups, the chi-squared test showed no significant difference in the difficulty of finding gluten-free, plant-based foods (X^2^ (16) = 24.1, *p* = 0.09). In contrast, Fisher’s exact tests revealed significant associations between age group and (i) time spent planning meals (*p* < 0.001) and (ii) cooking from scratch versus consuming processed foods (*p* = 0.004) when compared with before diagnosis. Patients ≥ 60 years more often reported spending ‘about the same amount of time’ on meal planning (51%) and making ‘no major changes’ to meals after CeD diagnosis (32%). Conversely, patients < 18 years more frequently reported spending ‘more time’ on meal planning (57%) and cooking from scratch (39%). Details on the answer distribution within all age groups are provided in Supplementary Tables S2 and S3.

## 4. Discussion

This cross-sectional questionnaire survey of a large population of patients with CeD investigated attitudes toward and barriers to adopting a more plant-dominant diet. The main findings were that more than half of the patients (56.8%) considered ‘eating more plant-based foods’, with health being the primary motivator. Our multiple regression analysis showed that age and sex were significantly associated with having considered eating more plant-based foods, whereas disease duration and prior dietetic counselling were not associated. Following diagnosis, over half of the participants reported changing their diet by a combination of more gluten-free products and more plant-based foods, while approximately one-third relied solely on gluten-free replacements. However, several barriers to adopting a more plant-dominant diet were identified. Most notably, ‘taste and texture’, ‘limited availability of gluten-free plant-based foods’, ‘nutritional concerns’, and cost were reported as barriers.

These findings suggest that patients with CeD generally have a positive attitude towards eating more plant-based foods; however, persistent barriers indicate that this perception may be influenced by the challenges of maintaining adequate dietary quality while following a GFD.

In the present study, disease duration showed only weak correlations with dietary habits (r = − 0.09 and r = − 0.27). Consistent with this, disease duration was not significantly associated with having considered eating more plant-based foods in the regression model. However, these patterns may reflect increasing familiarity with gluten-free dietary routines over time, potentially reducing the perceived practical burden of the diet. This interpretation is supported by a previous cross-sectional study showing that individuals with CeD who had followed a GFD for more than five years reported fewer negative emotions and practical difficulties than those who were more recently diagnosed [26]. Yet these behavioral adjustments appear to ease logistical challenges (e.g., planning and procuring meals) more than they address the qualitative aspects of the diet. This may explain why barriers such as taste and texture remain prominent, despite long-term adherence. Our results further support this interpretation. The factors most frequently reported as barriers, particularly taste/texture, the limited availability of gluten-free plant-based foods, and cost, were also the most influential drivers of patient’s dietary adaptation after diagnosis. This suggests that these barriers reflect the everyday constraints that shape dietary behavior, rather than the attitudinal resistance or lack of motivation among patients with CeD.

Age-related differences in dietary habits were also noted. Patients < 18 years more often reported spending additional time on meal preparation and cooking from scratch, whereas patients ≥ 60 years tended to maintain their usual meal planning routines after starting a GFD. These differences likely reflect the cumulative effects of time and experience on the learning process. Prior studies have similarly reported greater adherence difficulties among adolescents (13–17 years) and younger patients than older patients (≥60 years) with CeD [27,28]. Such age-specific differences provide a useful context for understanding variations in how patients adapt to a GFD and their capacity to make further dietary changes.

Although disease duration and age appear to influence dietary behavior in CeD, the nutritional composition of a GFD itself remains central to understanding the broader health implications. While gluten avoidance is essential for CeD management, it may carry potential metabolic risks. Industrially produced gluten-free products tend to have higher saturated fat, sugar, and salt content, and lower content of fiber and essential micronutrient, such as iron, calcium, and B vitamins [4,29]. These nutritional characteristics may contribute to dietary imbalances among patients who comply with the diet and, in turn, lead to cardiometabolic complications related to CVD [30].

It is important to note that the increased risk of CVD observed in patients with CeD [15] is likely to be multifactorial and may, at least in part, be caused by other risk factors, such as CeD-related systemic inflammation affected by dietary changes. Individuals with CeD have shown an increased CVD incidence despite generally favorable traditional risk profiles (e.g., lower BMI and lower smoking rates) [15]. Following this, post-diagnostic dietary changes associated with a GFD may be relevant as an additional cardiometabolic challenge and could help explain why an elevated CVD risk has been observed in this population.

Previous studies have identified unfavorable changes in cardiovascular risk factors after implementing a GFD, including increases in total and low-density lipoprotein cholesterol levels and BMI [10,31]. Retrospective studies have further shown a significantly higher prevalence of hypertension, hyperglycemia, hypercholesterolemia, and reduced high-density lipoprotein cholesterol levels in patients with CeD following a GFD [9,11]. A prospective observational study of newly diagnosed adults with CeD measured waist circumference, BMI, blood pressure, lipid profiles, and blood glucose levels. The authors found that these patients had a high risk of metabolic syndrome 1 year after starting on a GFD [32].

These findings collectively show that the cardiometabolic profiles of individuals with CeD following a GFD can worsen over time, highlighting the importance of examining how diet quality, and not only gluten exclusion, influences long-term health in CeD.

In this context, dietary patterns that incorporate more plant-based foods into a GFD may offer a potential strategy for mitigating the nutritional and metabolic risk factors associated with GFD. Evidence from the general population indicates that adherence to a plant-based diet (typically characterized by higher consumption of foods derived from plant sources and a lower consumption or total exclusion of animal-based foods) is associated with a lower risk of CVD and coronary heart disease [33,34]. Although these findings are non-CeD-specific, they provide a strong rationale for considering a more plant-dominant GFD. Importantly, any potential health benefits of a plant-dominant GFD are likely to depend on its nutritional quality. A dietary pattern centered on minimally processed plant foods, such as legumes, whole grains, fruits, and vegetables, may differ substantially in its metabolic impact from one characterized by refined gluten-free substitutes and ultra-processed plant-based products [21].

However, achieving a high-quality plant-dominant GFD in practice requires addressing behavioral and structural constraints. Patients often face barriers related to cost, individual preferences, limited dietetic counseling, and knowledge gaps [35,36]. A 2020 systematic review similarly identified insufficient patient education and low motivation as significant barriers to adherence to a GFD [36]. Collectively, these insights identify a plant-dominant GFD as a promising strategy for improving diet quality in patients with CeD. However, this potential requires attention to effective implementation through dietetic support and ensuring diet quality via in-depth nutritional assessment that extends beyond gluten avoidance.

A previous review noted that although current clinical practice guidelines are generally of low methodological quality, most have shifted toward more evidence-based approaches and identified dietitians as the primary professionals responsible for patient nutrition education [37]. Follow-up visits with a clinical dietitian aiming to educate and guide patients toward higher-quality plant-forward dietary choices within a GFD could help clarify misconceptions and prevent nutritional deficiencies in patients with CeD. Providing evidence-based resources, including recipes and practical strategies for incorporating more plant-based foods, may further support this.

Together, these interventions could reduce the perceived barriers related to taste, texture, the limited availability of gluten-free plant-based foods, and nutritional concerns, and not only improve dietary quality but also possibly reduce the associated cardiometabolic risk. They may also support long-term adherence and encourage positive attitudes toward a plant-dominant GFD.

### Strengths and Limitations

The strengths of this study are its large sample size and the use of a detailed questionnaire, which enabled a nuanced assessment of the attitudes toward and barriers to plant-based food consumption among individuals with CeD.

However, the generalizability of these results should be considered. Since the patients were members of the Danish Celiac Society, it may have introduced selection bias, as members often represent a more engaged and health-aware group of the celiac disease population. As a result, the findings may not fully reflect the broader population of individuals with celiac disease.

The response rate of approximately 34% (984 of 2861 invited) further introduces the possibility of non-response bias. As no information was available on non-respondents, we unfortunately cannot determine whether individuals who did not participate were less interested in dietary changes, more likely to experience survey fatigue, or differed demographically (e.g., sex, age).

As the questionnaire was distributed through the Danish Celiac Society, no objective dietary or clinical measures were available. Thus, this study is descriptive and focused on attitudes towards and perceived barriers to increasing plant-based food intake, rather than determining nutritional status.

Additionally, responses for patients aged < 18 years may be subject to informant bias, as they may partly reflect parental practices rather than the patients’ own. This should be considered when interpreting the observed age-related differences.

Although the questionnaire provided a definition of “plant-based foods,” patients may have still interpreted this term differently based on subjective understanding. Such variation in interpretation may have influenced how respondents evaluated motivations and barriers. Furthermore, existing literature does not provide clearly defined descriptions of what constitutes a “plant-based diet.” Several definitions exist, and some still allow for the inclusion of animal products to various degrees. As a result, the terminology often overlaps between what is described as a plant-dominant diet and a plant-based diet. This may introduce risk of misinterpretation for readers when comparing our survey terminology with other studies.

Additionally, the question “having considered eating more plant-based foods” was based on a single self-report item and may be subject to social desirability bias.

Validated instruments for assessing attitudes toward increasing plant-based food intake in individuals with celiac disease do not exist. This represents an important methodological gap, as the absence of validated measures limits comparability across studies and may reduce the precision with which such attitudes can be assessed.

## 5. Conclusions

In this study most patients expressed positive attitudes toward increasing their dietary intake of plant-based foods, with ‘health’ reported as the primary motivating factor, followed by ‘climate’ and ‘taste.’ However, practical, sensory and nutritional concerns appear to prevent the adoption of a more plant-dominant GFD. These findings reflect responses from a self-selected sample and should be interpreted in light of key limitations, including self-reported data, a non-validated questionnaire, and recruitment through a patient organization. As such, the results cannot be generalized to all individuals with celiac disease, but provide preliminary insight into factors that may shape dietary attitudes and perceived barriers, and offer emerging issues that warrant further investigation.

Although clinical outcomes were not assessed, interest in increasing plant-based food intake is noteworthy given the ongoing concerns about the nutritional and cardiometabolic risk profile of the typical gluten-free diet.

## 6. Future Directions

Given its descriptive design, this study was not intended to propose new theoretical frameworks but to provide foundational evidence that may guide future analytical or intervention-based research.

Additionally, as this study did not include objective measures on nutritional status, future interventional studies should incorporate validated dietary assessment tools and objective nutritional markers to evaluate actual intake and nutritional adequacy among individuals with celiac disease who are interested in increasing their intake of plant-based foods.

Furthermore, to improve representativeness, studies should also consider clinic-based or registry-based recruitments or mixed mode strategies, rather than relying solely on patient-organization distribution.

Therefore, future studies using validated instruments, objective measures and broader recruitment strategies are important directions, that can determine whether plant-dominant gluten-free dietary approaches centered on minimally processed plant-based foods, could support long-term nutritional quality and cardiometabolic health in this population.

## Figures and Tables

**Table 1:** Patient characteristics.

Characteristics		n (%)
**Age group** n total = 959 (100%)	<18 years	118 (12.3)
	18–29 years	90 (9.4)
	30–44 years	128 (13.3)
	45–59 years	322 (33.6)
	≥60 years	301 (31.4)
**Sex** n total = 959 (100%)	Female	782 (81.5)
	Male	177 (18.5)
**Disease duration** n total = 959 (100%)	0–1 years	71 (7.4)
	1–3 years	123 (12.8)
	3–5 years	124 (12.9)
	5–10 years	189 (19.7)
	>10 years	452 (47.1)
**Place of diagnosis** n total = 953 (99.4%)	Hospital	664 (69.7)
	Private practice specialist	120 (12.6)
	General practitioner	114 (12.0)
	No official diagnosis	39 (4.1)
	Other	16 (1.7)
**Referred to dietitian following diagnosis** n total = 949 (99.0%)	Yes	756 (79.7)
	No	193 (20.3)
**Other food allergies or intolerances besides celiac disease** n total = 948 (98.9%)	Yes	198 (20.9)
	No	706 (74.5)
	Don’t know	44 (4.6)

Percentages may not sum to 100% due to rounding.

**Table 2:** Self-reported dietary habits among patients with celiac disease.

Questions			n (%)
**How has your diet changed since being diagnosed with celiac disease?** n total = 946 (98.6%)	Gluten-free replacements		295 (31.2)
	More plant-based		90 (9.5)
	Combination		553 (58.5)
	Other		8 (0.8)
**How often is it difficult to find gluten-free food that fits your dietary preferences?** n total = 900 (93.8%)	Never		19 (2.1)
	Rarely		101 (11.2)
	Sometimes		323 (35.9)
	Often		361 (40.1)
	Always		65 (7.2)
	Don’t know		31 (3.4)
**How much time do you spend planning meals compared to the time spent before your diagnosis?** n total = 900 (93.8%)	Much less time		3 (0.3)
	Less time		4 (0.4)
	About the same		328 (36.4)
	More time		357 (39.7)
	Much more time		171 (19.0)
	Don’t know		37 (4.1)
**Has your consumption of food cooked from scratch versus processed food changed after your diagnosis?** n total = 897 (93.5%)	Much more from scratch		328 (36.6)
	More from scratch		295 (32.9)
	No major change		242 (27.0)
	More processed		28 (3.1)
	Much more processed		4 (0.4)
**Do you think you are getting enough of the following nutrients from your diet?** n total = 895 (93.3%)	Vitamins and minerals	Yes	647 (72.3)
		No	131 (14.6)
		Don’t know	117 (13.1)
	Wholegrain	Yes	374 (41.8)
		No	379 (42.3)
		Don’t know	142 (15.9)
	Dietary fibers	Yes	647 (72.3)
		No	163 (18.2)
		Don’t know	85 (9.5)
**Have you ever considered eating more plant-based foods?** n total = 892 (93.0%)	Yes		507 (56.8)
	No		385 (43.2)

Percentages may not sum to 100% due to rounding.

**Table 3:** Multivariable logistic regression model examining predictors of having considered eating more plant-based foods.

Predictor	OR	95% CI	*p*-Value
**Age (Overall)**	-	-	<0.001
**Sex (Female vs. Male)**	0.52	0.36–0.74	<0.001
**Disease duration (Overall)**	-	-	0.78
**Prior dietetic counselling (No vs. Yes)**	0.87	0.62–1.22	0.41

Full category-specific odds ratios for age groups are provided in Supplementary Table S1.

## Data Availability

A request for access to the data requires approval from the appropriate Danish authorities and is subject to regulations on personal data protection. Any requests for data should be sent to the corresponding author.

## Supplementary Materials

The following supporting information can be downloaded at https://www.mdpi.com/article/10.3390/nu18111673/s1. Figure S1: Distribution of prioritized factors influencing celiac disease patients’ choice to not follow a more plant-dominant diet while on a gluten-free diet. Patients selected their top three priorities, and bars indicate ranking of each item as patient’s 1st, 2nd, and 3rd priority. Y-axis represents the total percentage of patients who completed the ranking question (n = 507); Figure S2: Distribution of additional factors influencing celiac disease patients’ choice to follow a more plant-dominant diet while on a gluten-free diet. Patients selected their top three priorities, and bars indicate ranking of each item as patient’s 1st, 2nd, and 3rd priority. Y-axis represents the total percentage of patients who completed the ranking question (n = 507); Figure S3: Distribution of additional prioritized barriers toward eating a more plant-dominant diet while following a gluten-free diet among patients. Patients selected their top three priorities, and bars indicate ranking of each item as patient’s 1st, 2nd, and 3rd priority. Y-axis represents the total percentage of patients who completed the ranking question (n = 874); Figure S4: Distribution of responses according to additional factors influencing how patients adapted their current diet after celiac disase diagnosis. Patients selected their top three priorities, and bars indicate ranking of each item as patient’s 1st, 2nd, and 3rd priority. Y-axis represents the total percentage of patients who completed the ranking question (n = 904); Table S1: Multivariable logistic regression results showing odds ratios for all levels of age group, sex, disease duration, and prior dietetic counselling; Table S2: Answer distribution within age groups of time spent planning meals compared with before diagnosis; Table S3: Answer distribution within age groups of changes in cooking from scratch versus eating more processed foods after diagnosis.

## Abbreviations

The following abbreviations are used in this manuscript.

CeD	Celiac disease
GFD	Gluten-free diet
CVD	Cardiovascular disease
BMI	Body Mass Index
