# Supplementary material for: Attitudes and Barriers Toward Consumption of More Plant-Based Foods Among Danish Patients with Celiac Disease
Source: Nutrients. 2026 May 23;18(11):1673. doi: 10.3390/nu18111673 (PMC13259248; doi:10.3390/nu18111673)
Supplement: Supplementary file 1 [file nutrients-18-01673-s001.zip › nutrients-4305424-supplementary.pdf]

Supplementary material

Supplementary Figure S1: Distribution of prioritized factors influencing celiac disease patients' choice to not follow a more plant-dominant diet while on a gluten-free diet. Patients selected their top three priorities, and bars indicate ranking of each item as patient's 1st, 2nd, and 3rd priority. Y-axis represents the total percentage of patients who completed the ranking question (n=507).

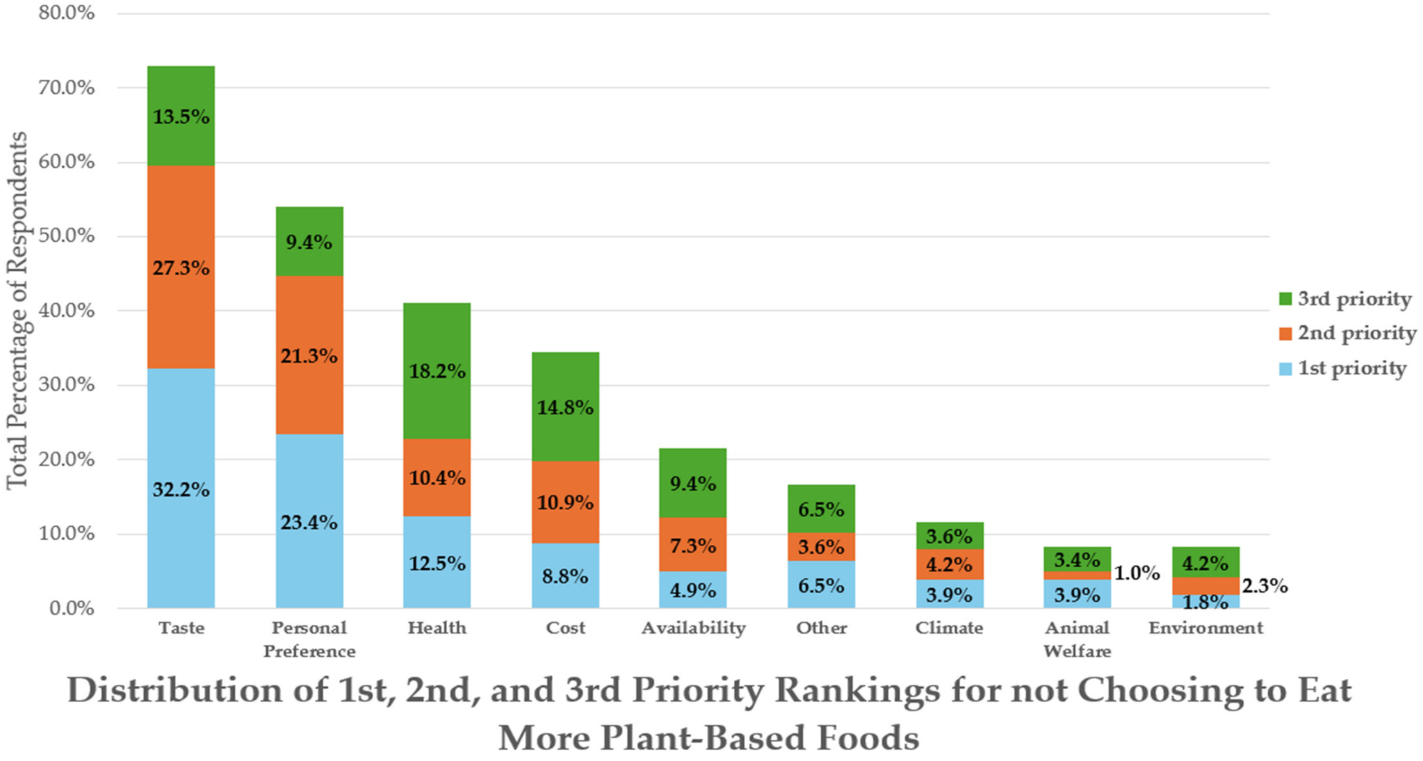

Supplementary Figure S2: Distribution of additional factors influencing celiac disease patients' choice to follow a more plant-dominant diet while on a gluten-free diet. Patients selected their top three priorities, and bars indicate ranking of each item as patient's 1st, 2nd, and 3rd priority. Y-axis represents the total percentage of patients who completed the ranking question (n=507).

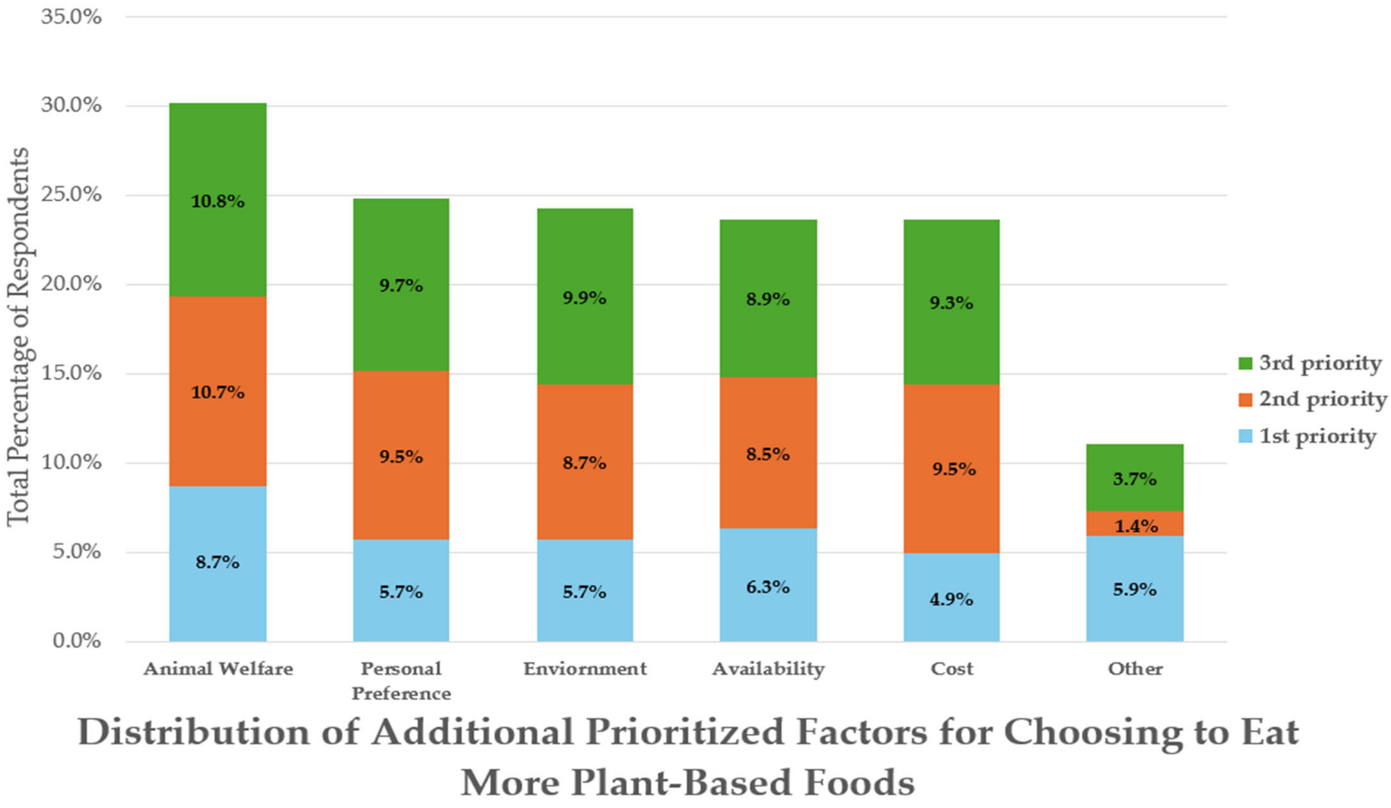

Supplementary Figure S3: Distribution of additional prioritized barriers toward eating a more plant-dominant diet while following a gluten-free diet among patients. Patients selected their top three priorities, and bars indicate ranking of each item as patient's 1st, 2nd, and 3rd priority. Y-axis represents the total percentage of patients who completed the ranking question (n = 874).

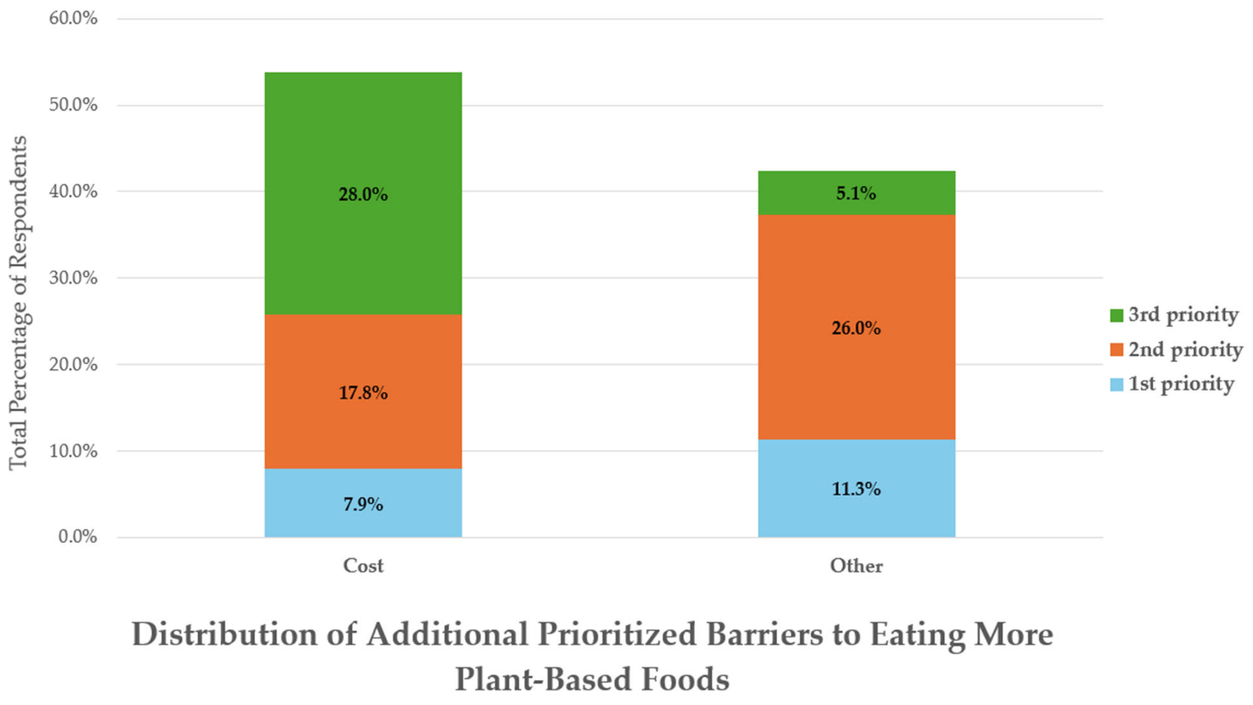

Supplementary Figure S4: Distribution of responses according to additional factors influencing how patients adapted their current diet after celiac disase diagnosis. Patients selected their top three priorities, and bars indicate ranking of each item as patient's 1st, 2nd, and 3rd priority. Y-axis represents the total percentage of patients who completed the ranking question (n = 904).

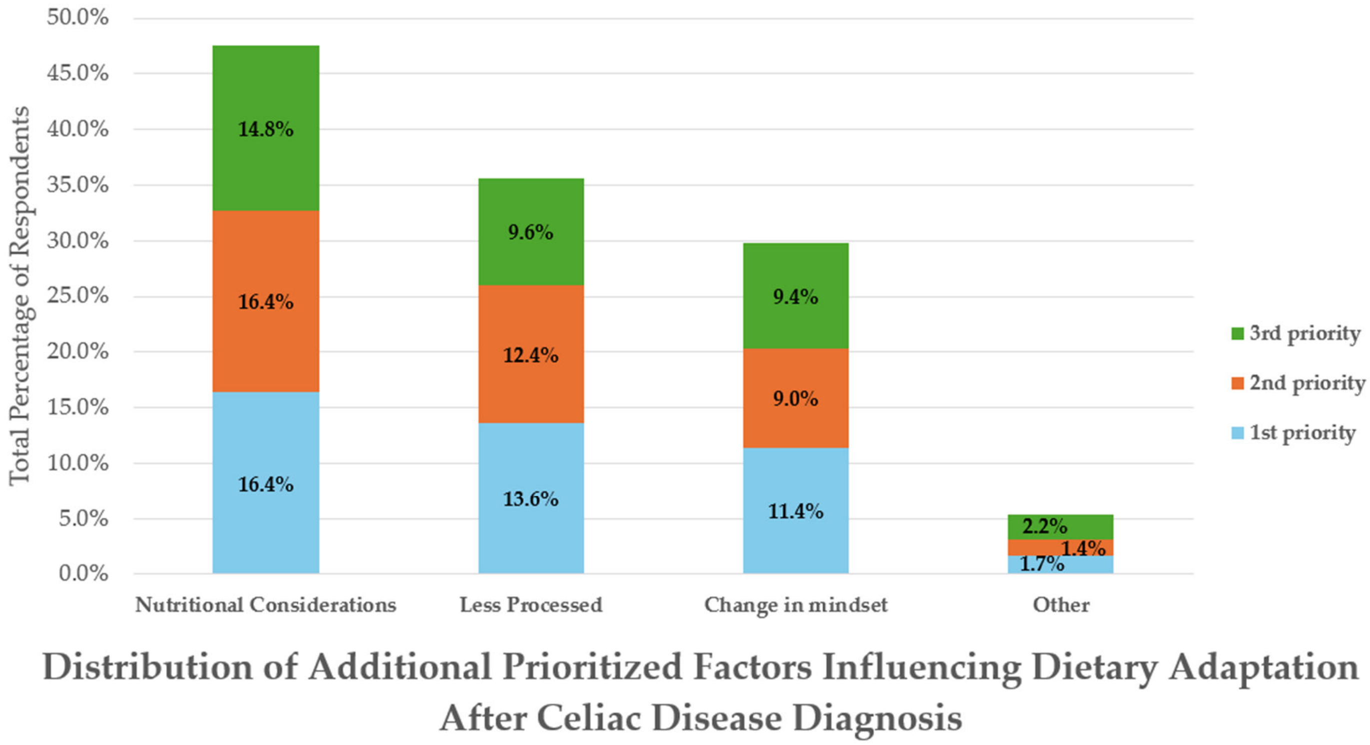

Supplementary table S1: Multivariable logistic regression results showing odds ratios for all levels of age group, sex, disease duration, and prior dietetic counselling.

Predictor	Category	OR	95% CI	p-value
**Age group, years**	<18 (ref)	-	-	-
	18-29	2.28	1.23-4.27	0.009
	30-44	4.72	2.62-8.67	<0.001
	45-59	2.79	1.70-4.63	<0.001
	≥60	2.40	1.44-4.05	0.001
**Sex**	Female (ref)	-	-	-
	Male	0.52	0.36-0.74	<0.001
**Disease Duration, years**	0-1 (ref)	-	-	-
	1-3	1.28	0.65-2.49	0.47
	3-5	1.12	0.58-2.17	0.74
	5-10	1.23	0.66-2.29	0.51
	>10	0.79	0.44-1.39	0.41
**Dietetic Counselling**	Yes (ref)	-	-	-
	No	0.87	0.62-1.22	0.41

Supplementary table S2: Answer distribution within age groups of time spent planning meals compared to before diagnosis.

Age group, years	Much less time, n (%)	Less time, n (%)	About the same, n (%)	More time, n (%)	Much more time, n (%)	Do not know, n (%)
**< 18**	1 (0.98)	0 (0.00)	13 (12.75)	58 (56.86)	28 (27.45)	2 (1.96)
**18-29**	0 (0.00)	0 (0.00)	19 (22.89)	26 (31.33)	28 (33.73)	10 (12.05)
**30-44**	1 (0.84)	0 (0.00)	31 (26.05)	48 (40.34)	32 (26.89)	7 (5.88)
**45-59**	1 (0.32)	1 (0.32)	118 (38.31)	131 (42.53)	49 (15.91)	8 (2.60)
**≥60**	0 (0.00)	3 (1.04)	147 (51.04)	94 (32.64)	34 (11.81)	10 (3.47)

Values are shown as n (%). Percentages are calculated within each age group.

Supplementary table S3: Answer distribution within age groups of changes in consuming foods cooked from scratch versus eating more processed foods after celiac disease diagnosis.

Age group, years	Much more from scratch, n (%)	More from scratch, n (%)	No major changes, n (%)	More processed, n (%)	Much more processed, n (%)
**< 18**	40 (39.06)	39 (39.61)	16 (15.84	6 (5.94)	0 (0.00)
**18-29**	31 (37.35)	36 (43.37)	13 (15.66)	3 (3.61)	0 (0.00)
**30-44**	49 (41.18)	39 (32.77)	24 (20.17)	5 (4.20)	2 (1.68)
**45-59**	93 (30.29)	107 (34.85)	98 (31.92)	8 (2.61)	1 (0.33)
**≥60**	115 (40.07)	74 (25.78)	91 (31.70)	6 (2.09)	1 (0.35)

Values are shown as n (%). Percentages are calculated within each age group.
